# Plasma levels of the MMP-9:TIMP-1 complex as prognostic biomarker in breast cancer: a retrospective study

**DOI:** 10.1186/1471-2407-13-598

**Published:** 2013-12-13

**Authors:** Stine B Thorsen, Sarah LT Christensen, Sidse Ø Würtz, Martin Lundberg, Birgitte S Nielsen, Lena Vinther, Mick Knowles, Nick Gee, Simon Fredriksson, Susanne Møller, Nils Brünner, Anne-Sofie Schrohl, Jan Stenvang

**Affiliations:** 1 Institute of Veterinary Disease Biology and Sino-Danish Breast Cancer Research Centre, Faculty of Health and Medical Sciences, University of Copenhagen, Strandboulevarden 49, DK-2100 Copenhagen, Denmark; 2 Olink Bioscience, Uppsala Science Park, Dag Hammarskjölds väg 52B, SE-75237 Uppsala, Sweden; 3 Innova Biosciences, Babraham hall, Cambridge CB22 3AT UK; 4 Danish Breast Cancer Cooperative Group, Rigshospitalet, Strandboulevarden 47, DK-2100 Copenhagen, Denmark

**Keywords:** Breast cancer, Plasma MMP-9:TIMP-1 complex, Proximity ligation assay, ELISA

## Abstract

**Background:**

Worldwide more than one million women are annually diagnosed with breast cancer. A considerable fraction of these women receive systemic adjuvant therapy; however, some are cured by primary surgery and radiotherapy alone. Prognostic biomarkers guide stratification of patients into different risk groups and hence improve management of breast cancer patients. Plasma levels of Matrix Metalloproteinase-9 (MMP-9) and its natural inhibitor Tissue inhibitor of metalloproteinase-1 (TIMP-1) have previously been associated with poor patient outcome and resistance to certain forms of chemotherapy. To pursue additional prognostic information from MMP-9 and TIMP-1, the level of the MMP-9 and TIMP-1 complex (MMP-9:TIMP-1) was investigated in plasma from breast cancer patients.

**Methods:**

Detection of protein:protein complexes in plasma was performed using a commercially available ELISA kit and, for the first time, the highly sensitive in-solution proximity ligation assay (PLA). We screened plasma from 465 patients with primary breast cancer for prognostic value of the MMP-9:TIMP-1 complex. Both assays were validated and applied for quantification of MMP-9:TIMP-1 concentration. In this retrospective study, we analyzed the association between the concentration of the MMP-9:TIMP-1 complex and clinicopathological data and disease free survival (DFS) in univariate and multivariate survival analyses.

**Results:**

Following successful validation both assays were applied for MMP-9:TIMP-1 measurements. Of the clinicopathological parameters, only menopausal status demonstrated significant association with the MMP-9:TIMP-1 complex; P = 0.03 and P = 0.028 for the ELISA and PLA measurements, respectively. We found no correlation between the MMP-9:TIMP-1 protein complex and DFS neither in univariate nor in multivariate survival analyses.

**Conclusions:**

Despite earlier reports linking MMP-9 and TIMP-1 with prognosis in breast cancer patients, we here demonstrate that plasma levels of the MMP-9:TIMP-1 protein complex hold no prognostic information in primary breast cancer as a stand-alone marker. We demonstrate that the highly sensitive in-solution PLA can be employed for measurements of protein:protein complexes in plasma.

## Background

Breast cancer is a frequently occurring malignancy, which in 2008 affected 1.38 million women worldwide [1]. In the treatment of breast cancer patients classical clinicopathological parameters together with estrogen (ER) and progesterone receptor (PR) and human epidermal growth factor receptor-2 (HER2) status are currently applied to stratify patients into high and low risk groups [2]. Presently, most high-risk breast cancer patients are offered systemic adjuvant therapy; however, a significant number of these patients are not in need of this treatment as they are cured by primary surgery and, in some cases, adjuvant radiotherapy [2,3]. Introduction of additional and validated prognostic biomarkers could add information to current risk stratifications resulting in a more effective management of future breast cancer patients.

Concentrations of both Matrix metalloproteinase-9 (MMP-9) and the naturally occurring Matrix metalloproteinase (MMP) inhibitor, Tissue inhibitor of metalloproteinases-1 (TIMP-1), have been investigated as tumor biomarkers in breast cancer [4-8]. MMP-9 belongs to the family of matrix degrading proteases, which play a role in both physiological and pathological tissue remodeling, including cancer growth and dissemination [9]. MMP-9 is primarily secreted as a pro-enzyme (pro-MMP-9), which can be activated to the mature enzyme (MMP-9) upon cleavage by proteinases [10]. In breast cancer high pre-operative serum MMP-9 concentration or MMP-9 activity in plasma have been suggested as prognostic markers indicating poor patient outcome [11-13]. Similar results have been reported for MMP-9 levels in tumor tissue extracts [14]. TIMP-1 counteracts the proteolytic effect of most MMPs, including pro-MMP-9 and MMP-9 [9]. Counter intuitively, high plasma levels of TIMP-1 have also been associated with a worse outcome in breast cancer patients [15]. Similar results have been reported for TIMP-1 levels in tumor tissue extract [4-6,16,17]. The observed association between high TIMP-1 levels and poor prognosis may be explained by the other functions that have been disclosed for TIMP-1: influence on cell growth [18,19], angiogenesis [20-22], apoptosis independently of its MMP-inhibitory functions [23-26], and on epithelial-mesenchymal transition [27].

The present study rests on the hypothesis that MMP-9:TIMP-1 complexes carry prognostic information when measured in plasma; i.e. we hypothesized that combining the prognostic association of MMP-9 and TIMP-1 by measuring their complex could potentially give additional prognostic information. Previous observations from tumor tissue have indicated that the fraction of TIMP-1 bound in complexes with other molecules is more closely related with a poor prognosis than the fraction of TIMP-1 present as a free molecule [28]. Since measurement of the complex between the two proteins has never been reported from breast cancer plasma, our study provides novel insight into the level of MMP-9:TIMP-1 and prognostic value in breast cancer plasma.

**Figure 1:**
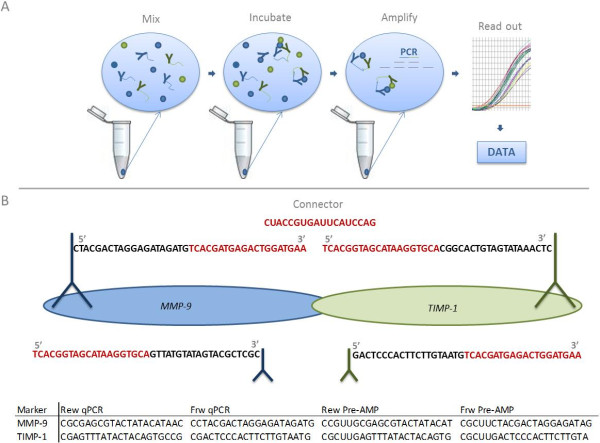
**Schematic outline of the Proximity Ligation Assay (PLA) technical procedure. A)** Schematic outline of the Proximity Ligation Assay (PLA) technical procedure. PLA probes directed against MMP-9 and TIMP-1 are incubated with the plasma sample allowing a binding of antibodies to target epitopes. Enzymatic ligations of the two oligonucleotide strands can be carried out only when the two PLA probes are in proximity, due to complex formation between MMP-9 and TIMP-1. Forming a PCR amplicon the antibody to antigen binding is now converted into a DNA strand, which can be amplified and later detected by real-time qPCR. **B)** Principle and sequential design of the MMP-9:TIMP-1 proximity probes. Each polyclonal antibody (MMP-9 and TIMP-1) has been divided into two pools, with one pool conjugated to the 5’ oligonucleotide (5’end) and the other pool conjugated to the 3’oligonucleotide (3’end). When mixing MMP-9 (3’end) probe with TIMP-1 (5’end) probe and plasma, the two probes will come into proximity, if their target antigens form a complex. The connector oligonucleotide is then used to connect the two oligonucleotide arms. Adding a ligase, the two arms will be ligated together, now forming the template of a PCR amplicon. The flanking 20 base pairs of the conjugated oligonucleotide represent the unique primer specific sequence, while the central part represents a universal sequence matching the connector oligonucleotide. The sequences of the specific primers are illustrated in the lower section of the figure. Rew: reverse primer, Frw: forward primer.

To oblige the potential of these protein biomarkers we focused on the necessities of assay optimization and verification of the potential biomarker. Since no validated assays for MMP-9:TIMP-1 complex determination in plasma have been published, we decided to apply two different antibody based techniques to measure the total amount of the complex (pro-MMP-9:TIMP-1 and MMP-9:TIMP-1). The MMP-9:TIMP-1 complex was quantified in preoperatively obtained plasma samples from 465 patients with primary breast cancer using a classical commercially available sandwich ELISA, which had not been validated for use with plasma samples by the supplier, and using the recently developed in-solution Proximity Ligation Assay (PLA, Figure 1). In brief, PLA employs two primary antibodies each linked by conjugation to a synthetic 40 nucleotide (nt) oligonucleotide. Oligonucleotides are designed with a specific sequence for primer targeting in the flanking 20 base pairs, while the central 20 base pair sequence is universal and specific for the connector. Upon simultaneous and proximal binding to a target protein the two oligonucleotides can be connected by ligation, and the oligonucleotide strand now forms a PCR amplicon detectable by quantitative real-time PCR.

Both assays performances were thoroughly validated with regard to recovery, linearity in plasma dilutions, and intra- and inter-variation. Except for menopausal status, no correlation of the MMP-9:TIMP-1 concentration was demonstrated with clinicopathological parameters. There was no relation between MMP-9:TIMP-1 and outcome neither in univariate survival analyses nor when combining the parameters in a multivariate analysis using the Cox proportional hazards approach with DFS as endpoint, suggesting that the MMP-9:TIMP-1 complex has no value as a stand-alone prognostic marker in breast cancer.

## Methods

### Patients

**Figure 2:**
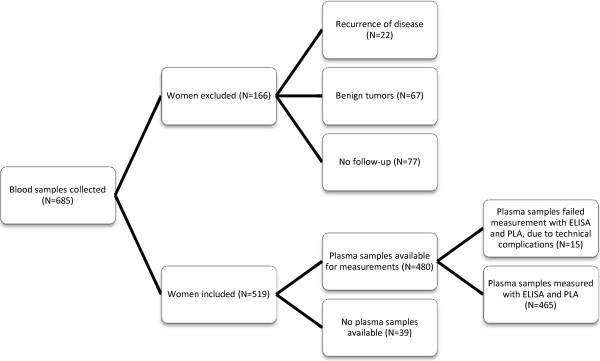
**Consort diagram of patients.** Schematic view of the distribution of plasma samples in the present study.

Preoperatively obtained EDTA plasma samples were obtained from women undergoing surgery (mastectomy or lumpectomy) with sentinel node procedure for primary breast cancer at Rigshospitalet, Copenhagen, Denmark. All patients were treated according to the current Danish Breast Cancer Cooperative Group Guidelines [3]. In the period 2001 to 2005, 685 samples were collected consecutively according to a standard operating procedure [15]. The present study was conducted as a retrospective study analyzing 465 plasma samples with both ELISA and PLA (Figure 2). The median age of the patients was 58 years (range of 38–80 years). Mean follow-up time was 2450 days (range 90–3360 days). The endpoint in the statistical survival analysis was disease free survival (DFS), defined as survival without recurrence, other malignancy, or death, when registered as the first event. Clinicopathological data registered for the patients were provided by the Danish Breast Cancer Cooperative Group (DBCG) and are summarized in Table 1. A total of 323 (69%) patients were postmenopausal. The lymph node status was known for all 465 patients of which 220 (47%) had lymph node-positive tumors. The histological types were divided between 80% ductal carcinomas, 15% lobular, and 5% other invasive cancers, reflecting a representative distribution of histological subtypes in the present cohort. The malignancy grade is only relevant for ductal or lobular carcinomas, and is consequently missing in patients with other invasive cancers. Totally, 134 patients had en event within 10 years after surgery (recurrence, other malignancy or death as the first event). For the multivariate analyses only 431 patients were included, due to missing clinicopathological data in 34 patients, and among these only 124 had an event. The study was conducted in compliance with the Helsinki II Declaration and written informed consent was obtained from all participants. The study was approved by the local ethical committee of Greater Copenhagen, approval number: H-4-2012-002. The REMARK Guidelines [29] were followed wherever applicable.

**Table 1:** Patient, tumor characteristics (N = 465) and association between MMP-9:TIMP-1 complex and the clinicopathological parameters

**Characteristics**	**ELISA**						**PLA**				
	**MMP-9:TIMP-1**						**MMP-1:TIMP-1**				
	**N**	**Q1(%)**	**Q2**	**Q3**	**Q4**	**P(χw**^**2**^** test)**	**Q1(%)**	**Q2**	**Q3**	**Q4**	**P(χw**^**2**^** test)**
Age						0.12					0.27
<50 years	107	30	16	25	21		26	27	24	16	
50-69 years	257	50	62	48	60		57	49	56	59	
≥70 years	101	20	22	27	19		17	24	20	26	
Menopausal status						0.03					0.03
Premenopausal	142	40	22	31	28		36	35	31	20	
Postmenopausal	323	60	78	69	72		64	65	69	80	
Tumor size					0.96						0.87
0-20 mm	302	66	66	64	64		63	67	63	66	
>20 mm	163	34	34	36	36		37	33	37	34	
Malignancy grade*						0.06					0.65
Grade 1	196	42	57	41	42		40	49	46	45	
Grade 2-3**	239	58	43	59	58		60	51	54	55	
Unknown	7										
Lymph node status						0.73					0.44
Positive	220	44	47	48	51		42	46	51	51	
Negative	245	56	53	52	49		58	54	49	49	
Hormone receptor status						0.50					.084
(ER and/or PR)								84			
Positive	378	79	86	83	80		82	16	84	80	
Negative	82	21	14	17	20		18		16	20	
Unknown	5										

*malignancy grade is only relevant in ductal or lobularcarcinoma, and therefore missing for 23 patients with other histological types.

**Grade 2: 140, Grade 3: 99.

Association between MMP-9:TIMP-1 complex and the clinicopathological parameters.

### Specimen characteristics

Blood samples were collected preoperatively following a standardized protocol [15]. The plasma samples were prepared by collecting blood in EDTA tubes, which were placed on ice immediately after sampling. The samples were centrifuged at 4000 *g* for 10 minutes and plasma was transferred to new tubes. Immediately hereafter the samples were stored at -80°C. The samples analyzed for TIMP-1:MMP-9 in this study had undergone from one to four freeze-thaw cycles before analysis.

### Assay methods

#### ***MMP-9:TIMP-1 analysis by ELISA***

Plasma concentrations of MMP-9:TIMP-1 complex were assayed using a commercially available ELISA (DuoSet® ELISA Development System, R&D Systems, R&D Systems Europe Ltd., United Kingdom; Human MMP-9:TIMP-1 complex catalog number DY1449) according to the manufacturer’s instructions. Before assaying plasma samples, the assay was validated for measurement of complexes in plasma. The validation process included investigations of reproducibility (intra-and inter-assay), recovery, and linearity upon dilution of samples. Recovery was determined by adding a fixed amount (1 ng/mL) of recombinant MMP-9:TIMP-1 complex to a plasma dilution series. Subsequently, recovery was calculated as the measured amount of complex in relation to the expected amount in each sample and reported in per cent. Intra-assay variation was determined by measurements of identical samples of a plasma pool diluted 3 times in reagent diluent buffer on the same plate. Inter-assay variation was determined by including duplicates of dilutions of the plasma pool on every plate measured. Linearity of signal was determined by measuring the signal in a dilution series of plasma (two-fold serial dilution ranging from 1-4000 times). Finally, the limit of detection was determined by repeated measurements of a blank sample and calculated as the mean of the signal in these blank samples plus three standard deviations. For validation purposes, a plasma pool obtained from a healthy donor plus a pool of plasma from cancer patients were used. For analysis, the plasma samples were diluted 3 times.

### MMP-9:TIMP-1 analysis by PLA

#### ***Proximity probe preparation***

The proximity probes directed against MMP-9 and TIMP-1 were prepared by linking a single batch of affinity purified polyclonal antibody (MMP-9 (R&D Systems, Cat.no. AF911) or TIMP-1 (R&D Systems, Cat.no. AF970)) to 3’-hydroxyl free and 5’-phosphate free 40-mer oligonucleotide sequences (Figure 1). Thereby, the unique amplicons are forming, which are representative for each target protein. The antibody-oligonucleotide conjugates were generated by Innova Biosciences (United Kingdom) using the Lightning-Link^TM^ technology. Conjugation quality was analyzed by SDS-PAGE.

#### ***Proximity ligation assay***

The basic PLA protocol has previously been described in details [30]. In brief, 2 μL of a plasma sample was mixed with 2 μL of plasma dilution buffer (Olink Bioscience, Sweden) including 200 pM of GFP as internal control standard spike-in. The mix was incubated at room temperature for 20 minutes. Probe mixture containing Probe Mix Diluent (Olink Bioscience), 1% BSA (Calbiochem/Merck, Germany) and 0.1% Triton X-100 (Merck, Germany) was added to each sample in a 1:1 ratio and incubated at 4°C overnight. Then 4 μL probed sample was mixed with 96 μL Ligation reaction buffer (Olink Bioscience, available upon request) containing 100 nM connector oligonucleotide and 0.0006 units of T4 DNA ligase (Fermentas, USA). The ligation was achieved by incubation at 37°C for 10 minutes, followed by 10 minutes of heat inactivation at 65°C. Prior to pre-amplification the connector oligonucleotide was digested using 1 unit of uracil-DNA excision mix (Epicenter, USA). Pre-amplification was performed in a total volume of 25 μl by mixing 20 μl of the ligated product with 5 μL PCR mix (1× PCR buffer (Invitrogen, Denmark), 15 mM MgCl_2_ (Invitrogen), 1 mM dNTP (Invitrogen), 0.2 μM of each forward and reverse pre-amplification primer (Figure 1) (Biomers, Germany), and 7.5 units Platinum Taq polymerase (Invitrogen)), using the same amplification protocol as previously described [30]. Prior to qPCR, the products were diluted 5-fold in 1× Tris-EDTA buffer.

qPCR was performed on a LightCycler 480 (Roche, Denmark) using a 384-well format. The diluted DNA products were mixed with 1.4× Fast Universal Master Mix (Life Technologies, Denmark), dH_2_O and 0.05 units of uracil-DNA excision mix (Epicenter) and incubated for 30 minutes at 37°C to digest any leftover primers in the solution. Seven μL of each sample was then transferred to each well and mixed with 3 μL of 3 μM primer (Figure 1) (Biomers), dH_2_O and 0.8 μM TaqMan probe (Life Technologies, Denmark) to a total sample volume of 10 μL per well. The thermal cycler program was initiated with 5 min at 95°C followed by 45 cycles of 15 s at 95°C and 1 min at 60°C. The time period from initiation of PLA measurements until end of all incubations was four weeks.

#### ***Validation of the MMP-9:TIMP-1 proximity ligation assay***

We initiated our validation of the PLA technique as a tool for measuring protein:protein complex in biological samples with some basic experiments of protocol optimization. The performances of the MMP-9 and TIMP-1 probes were assessed by their ability to measure increasing concentration of the recombinant proteins. MMP-9 (R&D Systems, Cat.no. 911-MP-010), TIMP-1 (in-house, purified as [31]), and complexed MMP-9 and TIMP-1 in PBS + 0.1% BSA were tested. Probe concentrations of 50, 75, 100 and 200 pM were tested in order to establish the optimal assay setup, which gave the lowest background signal and best linear range for each of the two possible probe combinations. The incubation time and temperature were tested in two different settings, one hour at 37°C and overnight at 4°C. The effect of pre-amplification was tested in both buffer and plasma to explore if a less time-consuming protocol could be applied. Unspecific annealing of the qPCR primers was tested by incubation with the MMP-9:TIMP-1 probes and recombinant protein, followed by qPCR on the resulting amplicon with primers for the total MMP-9, total TIMP-1 or specific primers for the MMP-9:TIMP-1 complex as positive controls. The dynamic range of the MMP-9:TIMP-1 complex measurements in human plasma was evaluated by selecting four different plasma samples from breast cancer patients. Using the ELISA measurements we selected two samples with a low level of TIMP-1 and two with a high level of TIMP-1. This was done to investigate if the decrease in signal was correlated with the dilution factor, but also to find the optimal dilution range of the plasma samples. The precision of the assay was investigated by analysis of these four samples, four times, on four different days. Every day, the samples were handled independently and the standard curves were freshly made. The measurements were made in quadruples, separated prior to the qPCR. This gave an estimate of the intra-assay variation within a run on the qPCR plate. The measurement between days allowed for an estimate of the inter-assay variation of the assay. For analysis, the plasma samples were diluted 50 times.

#### ***Preparation of internal standards, standard curves, and samples for recovery studies for the proximity ligation assay***

For internal control standard we used recombinant green fluorescent protein (GFP) (Vector Laboratories, USA), which was spiked in the plasma dilution mix (Olink Bioscience) and thereby added to all sample incubations. GFP was diluted in PBS + 0.1% BSA (Calbiochem/Merck, Denmark) and mixed with the plasma diluent mix to a final concentration of 10 nM. We used the data from GFP to evaluate the technical performance of the PLA runs. A standard curve of the MMP-9:TIMP-1 complex was made to investigate the linear range of the assay. Also, the standard curve was used for calculation of the MMP-9:TIMP-1 concentration of each sample. The recombinant MMP-9 and TIMP-1 were mixed in PBS + 0.1% BSA to create the complex standard curve; this ranged from 0.010 to 10 nM. The standard curve was prepared and went through a PCR run and then divided into aliquots and stored at -20°C until qPCR run; these standard curves were loaded to each qPCR plate. Recovery and specificity studies were made in both PBS + 0.1% BSA, chicken plasma (GeneTex, USA, Cat.no. GTX73211) and human plasma. Recombinant proteins were spiked in these three different materials in the range of 200 pM to 2000 pM.

### Statistical analysis

For analysis of association between the level of MMP-9:TIMP-1 and the clinicopathological parameters and DFS; defined as survival without recurrence, other malignancy, or death, when registered as the first event, patients were divided into four groups of 25% quartiles (Q1: 0-25%, Q2: 25-50%, Q3: 50-75%, Q4: 75-100%) of equal size according to increasing MMP-9:TIMP-1 level. This was done similarly for both ELISA and PLA measurements. Associations between the clinical parameters and MMP-9:TIMP-1 levels were tested by χ^2^tests. Correlation between ELISA and PLA measurements were analysed by Pearson correlation coefficient and by estimation of the concordance. The Kaplan-Meier method was used to estimate survival probabilities in the univariate survival analysis and the groups were compared by the log rank test. The Cox proportional hazards model was used for multivariate analysis, including ELISA measurements of MMP-9:TIMP-1 and all the clinicopathological parameters: age , menopausal status, tumor size, lymph node status, hormone receptor status and malignancy grade. For parameters having more levels, they were all included in the test for significant effect. Patients with missing values were excluded from the calculations. P values less than 5% were considered significant. The SAS software package was used to analyse the data.

## Results

### Validation of MMP-9:TIMP-1 ELISA

When performing the MMP-9:TIMP-1 ELISA we obtained a standard curve in accordance with the datasheet from the manufacturer. The standard curve covers the range 0.05 ng/mL to 3 ng/mL; however, based on determination of background signal the functional lower limit of detection (background signal plus three standard deviations) was 0.169 ng/mL. Validating the assay, we found linearity of the signal in plasma diluted up to four times. In addition, we found that the assay performed well with regard to recovery of signal when plasma samples were diluted 0–4 times as we determined a recovery between 98–116%. Finally, the intra- and inter-assay variations were 4.7% (N = 16) and 19.9% (N = 24), respectively.

### Validation of MMP-9:TIMP-1 PLA

**Figure 3:**
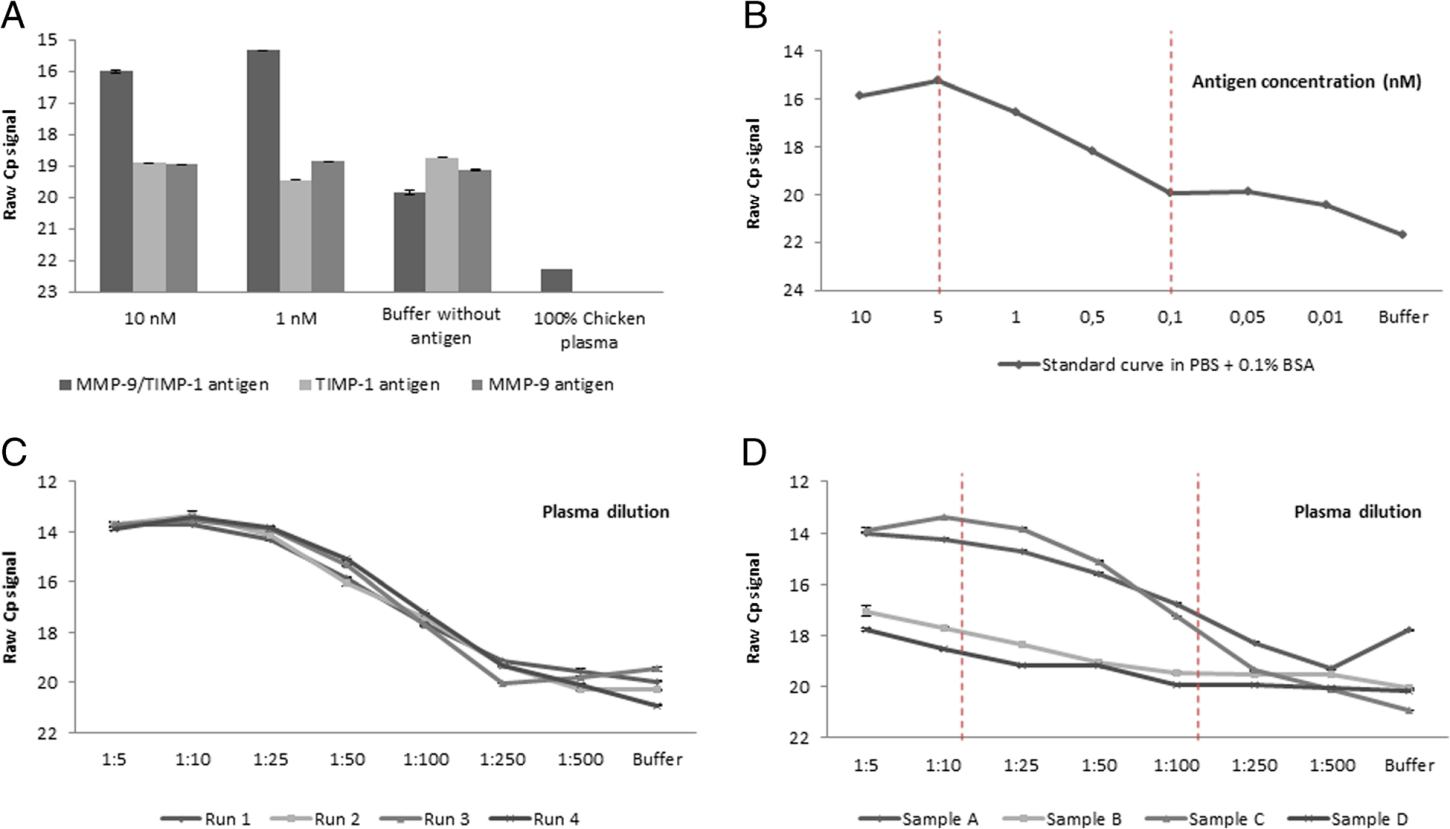
**Performance of the MMP-9:TIMP-1 proximity ligation assay. A)** The dark grey bar represents the specific signal from buffer when both MMP-9 and TIMP-1 antigens are present. The light grey bar represents the unspecific signal from a buffer sample with only MMP-9 as spike-in, while the medium grey bar represent the unspecific signal from a buffer sample with only TIMP-1 as spike-in. For all spike-in antigens the concentration stated on the x-axis applies. Further, the cross-reactivity in chicken plasma was demonstrated to be below buffer level. Values are reported as raw Cp signals. **B)** Standard curve for the MMP-9:TIMP-1 complex in PBS + 0.1% BSA buffer. The TIMP-1 (5’probe) and MMP-9 (3’ probe) were incubated with increasing amount of the MMP-9 and TIMP-1 antigens. This curve demonstrates the performance of the assay by assessing linear range in buffer settings and thereby the correlation between dose and Cp signal. The linear range is illustrated by the dotted lines and values are reported as raw Cp values. **C)** Inter-assay variation was determined by measuring the same breast cancer plasma sample on four different days. The four lines represent the sample measured on four different days (run 1, 2, 3 and 4). Values are reported as raw Cp signals. **D)** Plasma dilution curves of the MMP-9:TIMP-1 complex from four different breast cancer patients. Samples A and C have a high level of TIMP-1 protein, while samples B and D have a low level of TIMP-1 protein. The combined linear range was set between the 1:10 and 1:100 dilutions, illustrated by the dotted lines. Values are reported as raw Cp signal. Error bars represent standard deviation.

The sensitivity was not enhanced with increasing probe concentration and 50 pM was found to be the optimal probe concentration (Additional file 1: Figure A). The complex was measured satisfactorily with both combinations of the probes. In the analysis of the 465 plasma samples the MMP-9 (5’phosphate) and TIMP-1 (3’hydroxyl) probe combination was used due to a slightly better linear range (Additional file 1: Figure B). The sensitivity of the assay was increased with 1–2 Cp values by leaving the samples to incubate with the proximity probe mix over night at 4°C (Additional file 1: Figure A). Efficiency of the pre-amplification was demonstrated by analyzing a plasma dilution curve with and without pre-amplification, which increased the Cp-value with 15 points (Additional file 1: Figure A). Thus, the extra variation potentially introduced by the additional step in the PCR did not outdo the robustness gained by pre-amplification. This procedure was investigated in one breast cancer plasma sample with low and one with a high level of TIMP-1, determined by ELISA in a previous study [15], and it was found that implementation of pre-amplification was especially important when analyzing samples with low levels of target (data not shown). Signals from unspecific annealing of the qPCR primers were low and did not interfere with the specific signal, demonstrating high specificity of the primers (Additional file 1: Figure A). To assess linearity of signal and assay performance a standard curve for the complex in buffer including the whole linear range was performed (Figure 3). After measuring two plasma samples with low and two with high TIMP-1, a joint linear range for both low and high expressing samples was found between the 1:10 and 1:100 dilutions (Figure 3). A concentration dependent signal was observed only when both MMP-9 and TIMP-1 antigens were present in the buffer and could form the MMP-9:TIMP-1 complex, demonstrating the high specificity of the assay. Further, no unspecific signal and no signal above background were produced when analysing a chicken plasma sample (Figure 3).

The recovery was investigated in human control plasma and was found to be in the range of 80-103%. The precision and performance of the MMP-9:TIMP-1 proximity assay was investigated by analysing four breast cancer plasma samples for their levels of MMP-9:TIMP-1 complex and the GFP spike-in. Both the intra-variation within one plate and the inter-variation between plates were calculated. The overall intra-variation was 2.5% (range: 0.8-6.8% (median: 1.9%)) (N = 16) for the MMP-9:TIMP-1 measurements in breast cancer plasma, while the inter-variation between plates was 19.9% (range: 11.3-29.2% (median: 19.6%)) (N = 16). The variation is given in CV% based on linearized data. The whole dilution curve was assessed for each sample, however, only the area corresponding to the final area of measurements for the 465 plasma samples was used when calculating the variation, which for the MMP-9:TIMP-1 complex is the 1:50 dilution (Additional file 1: Figure C). Furthermore, these data also demonstrate that 1–4 freeze/thaw cycles, which corresponds to the number of cycles for the analysed breast cancer samples, do not affect the levels of MMP-9:TIMP-1 complex, thereby eliminating the concern of complex degradation upon repeated freeze/thaw cycles.

### Comparison of ELISA and PLA methods

Including all 465 samples, the correlation between the two technical approaches was examined. A Pearson correlation coefficient of 0.53 (Additional file 2) with a P-value of <0.001 was found. Thus, sample measurements demonstrated only a low level of correlation between the two molecular techniques.

### Analysis of EDTA plasma from breast cancer patients and association to clinicopathological variables

The MMP-9:TIMP-1 complex was measured with ELISA and with PLA in plasma samples obtained preoperatively from 465 breast cancer patients (Figure 2). The mean level of MMP-9:TIMP-1 measured by ELISA in the 465 breast cancer plasma samples was 3.63 ng/mL (0.11 – 14.77 ng/mL) and the mean level of MMP-9:TIMP-1 measured by PLA in the 465 breast cancer plasma samples was 0.35 nM (0.09 – 3.50 nM).

The association between the MMP-9:TIMP-1 complex and the clinicopathological parameters is summarized in Table 1 for both the ELISA and PLA measurements. Menopausal status was significantly associated with MMP-9:TIMP-1 complex concentration measured by both ELISA (P = 0.03) and PLA (P = 0.028). No other clinicopatological parameters were associated with the plasma MMP-9:TIMP-1 complex (Table 1).

### Univariate survival analysis

**Figure 4:**
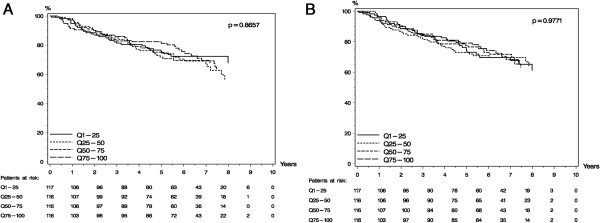
**Univariate survival analysis of disease free survival (DFS) in plasma for all 465 patients. A)** MMP-9:TIMP-1 measured by ELISA. **B)** MMP-9:TIMP-1 measured by PLA. Patients are divided into four groups of equal size (Q1-Q4) according to increasing plasma MMP-9:TIMP-1 levels; Q1 being the group with the lowest level.

For survival analysis patients were divided into the four quartiles as described above. When constructing a Kaplan-Meier curve to provide insight into the shape of the survival function, plasma MMP-9:TIMP-1 complex as determined by ELISA did not significantly predict a shorter or longer DFS (P = 0.8657) (Figure 4A). In concordance with the ELISA measurements, the plasma MMP-9:TIMP-1 complex as determined by PLA did not significantly predict a shorter or longer DFS (P = 0.9771) in a Kaplan-Meier analysis (Figure 4B). Other prognostic factors that were significantly associated with DFS in the univariate analyses were age (P < 0.0001), tumor size (P = 0.0205), malignancy grade (P = 0.0300), menopausal status (P = 0.0104), and hormone receptor status (P = 0.0034), which is in accordance with previous findings [15].

### Multivariate survival analysis

All the clinicopathological parameters and all ELISA measurements of MMP-9:TIMP-1 were included in the analysis using the expected best prognostic outcome as base line for each parameter. From the Cox proportional hazards analysis neither the ELISA measurements of MMP-9:TIMP-1 (Q2, Q3, or Q4) nor menopausal status, tumor size, lymph node status, or malignancy grade were associated with DFS. However, age (≥ 70 years) and hormone receptor status (negative) were associated with shorter DFS (P < 0.0001, hazard ratio (HR) = 2.48, 95% Confidence interval (CI) = 1.63 – 3.77; P = 0.002, hazard ratio = 0.47, 95% CI = 0.30 – 0.75), respectively (Table 2). An identical multivariate analysis was performed employing PLA measurements of the MMP-9:TIMP-1 complex. From the Cox proportional hazards analysis neither the PLA measurements of MMP-9:TIMP-1 (Q2, Q3, or Q4) nor menopausal status, tumor size, lymph node status, or malignancy grade were associated with DFS. However, also in this model age (≥ 70 years) and hormone receptor status (negative) were associated with shorter DFS (P < 0.0001, HR = 2.49, 95% CI = 1.64 – 3.78; P = 0.002, HR = 0.48, 95% CI = 0.30 – 0.76), respectively (Table 2). HRs and CIs for the MMP-9:TIMP-1 (PLA) measurements demonstrate a tendency towards a continuous decrease in HR with higher complex values, though at no significant level.

**Table 2:** Multivariate analysis using Cox proportional hazards model on DFS, including only patients with no missing values (N = 431)*

**Parameter**	**ELISA**			**PLA**		
	**HR**	**95% Cl**	**P**	**HR**	**95% Cl**	**P**
MMP-9:TIMP-1 complex						
Q2 vs. Q1	1.08	0.65-1.80	0.771	0.91	0.56-1.49	0.712
Q3 vs. Q1	1.12	0.68-1.85	0.652	0.84	0.51-1.38	0.493
Q4 vs. Q1	1.06	0.64-1.77	0.812	0.76	0.46-1.27	0.294
Age						
50-69 years vs. < 50 years	0.82	0.39-1.71	0.592	0.82	0.39-1.72	0.601
≥ 70 years vs. < 50 years	2.48	1.63-3.77	<0.0001	2.49	1.64-3.78	<0.0001
Menopausal status						
Post- vs. premenopausal	1.14	0.58-2.27	0.705	1.19	0.60-2.36	0.618
Tumor size						
>20 mm vs. 0–20 mm	1.25	0.85-1.82	0.254	1.24	0.85-1.81	0.261
Lymph node status						
Positive vs. negative	1.22	0.84-1.77	0.301	1.26	0.87-1.84	0.22
Hormone receptor status						
Positive vs. negative	0.47	0.30-0.75	0.002	0.48	0.30-0.76	0.002
Malignacy grade						
Grade 2 + 3 vs. Grade 1	1.15	0.76-1.75	0.499	1.14	0.75-1.73	0.536

*34 patients are excluded from the analysis due to missing values for one or more of the prognostic factors.

## Discussion

We validated and applied two different MMP-9:TIMP-1 assays and found that both the commercially available ELISA and the PLA reliably quantified the MMP-9:TIMP-1 complex concentration in plasma samples from breast cancer patients. In particular, we report for the first time that in-solution PLA can be used for quantification of protein:protein complexes in plasma. Except for menopausal status, no associations between the MMP-9:TIMP-1 concentration and clinicopathological parameters were found. Further, there was no relation between MMP-9:TIMP-1 and outcome when combining the parameters in a multivariate analysis, suggesting that the MMP-9:TIMP-1 complex has no value as a stand-alone prognostic marker in breast cancer.

Both assays applied were thoroughly validated prior to analysis of plasma samples. Thus, we ensured performance with regard to recovery, linearity in plasma dilutions, and intra- and inter-variation. Moreover, for the PLA, we performed specificity experiments using recombinant antigen solutions with or without the specific antigens present.

Results obtained by the two different MMP-9:TIMP-1 complex assays were weakly correlated (Pearson correlation coefficient 0.53, P < 0.001). However, due to different procedures in the protocols and the fact that different antibodies are used in the two technical systems, some variation between ELISA and PLA measurements is expected. Use of different antibodies, in particular polyclonal antibodies, makes possible identification of various conformations and, potentially, of different complexes with third components. However, the overall statistical output was very similar for the two techniques. Use of the PLA method offers increased sensitivity when compared to conventional ELISAs; still, despite increased sensitivity no association with outcome was found. It should be noted that previous data on co-precipitation of TIMP-1 and MMP-9 have demonstrated that antibodies do not interfere with the complex formation [32].

Measurement of the MMP-9:TIMP-1 protein:protein complex concentrations in plasma from breast cancer patients for prognostic purposes has not previously been described although numerous publications have indicated that both molecules, when measured individually, are indicative of patient prognosis [5,6,11,15]. Moreover, a previous study suggested that when analyzing TIMP-1 in breast cancer tissue, it is the fraction of TIMP-1 in complex with other molecules that is associated with poor prognosis [28]. In that study, TIMP-1 present in tumor tissue in an unbound form appeared not to be related with a poor outcome, whereas increasing amounts of complex-bound TIMP-1 was related with a shorter recurrence-free and overall survival. This relation could not be confirmed in serum samples, however, the study included a limited number of samples and was weakened by a number of technical issues [33]. Therefore we addressed this by studying the complex of TIMP-1 and pro-MMP-9/MMP-9 and in the present study of 465 breast cancer patients we were not able to find support for the hypothesis that the concentration of MMP-9:TIMP-1 complexes is indicative of prognosis in breast cancer patients. It could be speculated that the complexes in plasma are not necessarily related to those detected in tumor tissue. It has been shown that total TIMP-1 levels in plasma and tissue extracts from breast cancer patients are only weakly correlated [34], and the current findings suggest that the same holds true for TIMP-1 complexes. In the study from 2008, it was concluded that tissue-related TIMP-1 does not gain access to the blood stream proportionally with its level in the tumor and as such plasma TIMP-1 is not a surrogate marker for tissue TIMP-1. Assuming that complex-bound TIMP-1 in tissue carries prognostic information, it appears likewise that plasma MMP-9:TIMP-1 is not a surrogate marker for complexes present in tissue. It can be speculated that a fraction of the MMP-9, TIMP-1 and complexes present in tissue are captured or degraded in the tumor and therefore never reach the circulation.

However, it should be noted that despite significant associations between classical prognostic parameters (age, tumor size, malignancy grade, hormone receptor status, menopausal status) and DFS in univariate analysis, only age and hormone receptor status remained significant in the multivariate analyses. It could be speculated that this is partly due to the fact that all the high-risk patients (N = 333) received adjuvant systemic therapy. Consequently, the outcome for these patients is likely to be positively affected by the therapy and the result of our analyses may be biased.

On the assumption that previous findings hold true, i.e. that MMP-9 and TIMP-1 when measured individually in plasma are related with prognosis, it follows from our data that complexes between the two molecules do not have the same prognostic value. One reason for this could be the complex binding biology of these two protein molecules. TIMP-1 binds both pro-MMP-9 and mature MMP-9 in a 1:1 stoichiometry [28,29,35]. However, a vast amount of different molecules may bind to TIMP-1 (e.g. most of the MMPs) and to MMP-9 (e.g. the TIMP-1, -2, -3 and -4); this implies that the complex formation is not necessarily solely dependent of free MMP-9 and TIMP-1 and that numerous factors can affect the formation of the MMP-9:TIMP-1 complex [9,36].

Several functional implications of capturing TIMP-1 as well as MMP-9 in a complex in plasma could be envisioned. High levels of both total MMP-9 and total TIMP-1 have been shown to correlate with adverse prognosis and accordingly, it could be speculated that complexes consisting of the two proteins would be related to prognosis in a similar way. A functional role for the complex could also be imagined, e.g. as a carrier complex or as an aid in protecting both proteins from degradation. Conversely, complex formation could also be regarded as a potential mechanism for removal of free MMP-9 and TIMP-1 from plasma. Hence, functional considerations do not point to a simple biological role for the complex.

## Conclusions

In conclusion, we have thoroughly validated and employed two antibody-based assays for measurement of MMP-9:TIMP-1 complexes in plasma. Our data support future use of the highly sensitive, low sample-consuming PLA for detection of protein:protein complexes in plasma. We find that despite the previously described prognostic impact of plasma MMP-9 and TIMP-1 when measured individually, the complex consisting of these two proteins is not related with prognosis in this cohort of breast cancer patients, thus pointing to another biological role of MMP-9:TIMP-1 complexes in breast cancer patients.

## Abbreviations

Ab: Antibody; Ag: Antigen; Cp: Crossing point; CI: Confidence interval; CV: Coefficient of variation; DFS: Disease free survival; ELISA: Enzyme-linked immunosorbent assay; GFP: Green fluorescent protein; HR: Hazard ratio; mAb: Monoclonal antibody; MMP-9: Matrix metalloproteinase-9; nt: Nucleotides; pAb: Polyclonal antibody; PLA: Proximity ligation assay; qPCR: Quantitative real-time polymerase chain reaction; RT: Room temperature; TIMP-1: Tissue inhibitor of metalloproteinase-1.

## Competing interests

Martin Lundberg and Simon Fredriksson are employees at Olink Bioscience AB owner of intellectual property on the proximity ligation assay.

## Authors’ contributions

SBT, JS, NB and AS conceived of the study and coordinated activities. SF developed the in-solution PLA, ML optimized the assay. MK and NG optimized the conjugation step in the probe preparation process. SBT and SLTC performed PLA validation and measurements in plasma samples and interpreted results with help from JS. BSN and SØW performed ELISA validation and measurements in plasma samples and interpreted results with supervision from AS. LV purified the recombinant TIMP-1 protein employed as a standard in the PLA. SM performed all statistical analyses. SBT, SLTC, JS and AS wrote the manuscript with help from SM and NB. All authors read and approved the final manuscript.

## Authors’ information

Anne-Sofie Schrohl and Jan Stenvang shared senior authorship.

## Pre-publication history

The pre-publication history for this paper can be accessed here:

http://www.biomedcentral.com/1471-2407/13/598/prepub

## Supplementary Material

Additional file 1 ***Validation of MMP-9:TIMP-1 PLA.*** Figure A. Validation of MMP-9:TIMP-1 assay performance. A) Various probe concentrations. Four different proximity probe concentrations (50, 75, 100 and 200 pM) were incubated with plasma in different dilutions. The probe concentration of 50 pM demonstrated the best linear range and this was evaluated as being the optimal protocol. B) Sample and probe incubation. The difference between incubation of sample and probe mix for one hour at 37°C and ON at 4°C was tested. An increased sensitivity of 1–2 Cp values was assessed by ON incubation. C) Test for pre-amplification efficiency. To investigate if a pre-amplification was increasing the signal in the qPCR, a standard curve was run with and without pre-amplification. An increase of 15 Cp values was demonstrated due to pre-amplification illustrating high efficiency. D) Unspecific annealing of the qPCR primers. The interaction of free pre-amplification primers on the true signal was evaluated. Primer number 5 corresponds to MMP-9 and primer number 7 corresponds to TIMP-1. Only the correct 7–5 primer combination gave a true signal. The three other combinations gave only background signals. Figure B Difference between detecting the MMP-9:TIMP-1 complex with the MMP-9 (5’phosphate) and TIMP-1 (3’hydroxyl) probe combination versus the TIMP-1 (5’phosphate) and MMP-9 (3’hydroxyl) probe combination. It was found that the MMP-9 (5’phosphate) and TIMP-1 (3’hydroxyl) probe combination was performing slightly better. Figure C Dilution curves of plasma samples A-D analysed by PLA in four different days for the MMP-9:TIMP-1. These curves were used to calculate both intra- and inter-assay variation. Click here for file

Additional file 2 ***Comparison of ELISA and PLA methods.*** Pearson correlation between MMP-9:TIMP-1 (ELISA) and MMP-9:TIMP-1 (PLA). Click here for file
