# Supplementary figures and images for: Plasma levels of the MMP-9:TIMP-1 complex as prognostic biomarker in breast cancer: a retrospective study
Source: BMC Cancer. 2013 Dec 13;13:598. doi: 10.1186/1471-2407-13-598 (PMC3878682; doi:10.1186/1471-2407-13-598)

Additional file 1, Figure A

Additional file 1, Figure B

Additional file 1, Figure C


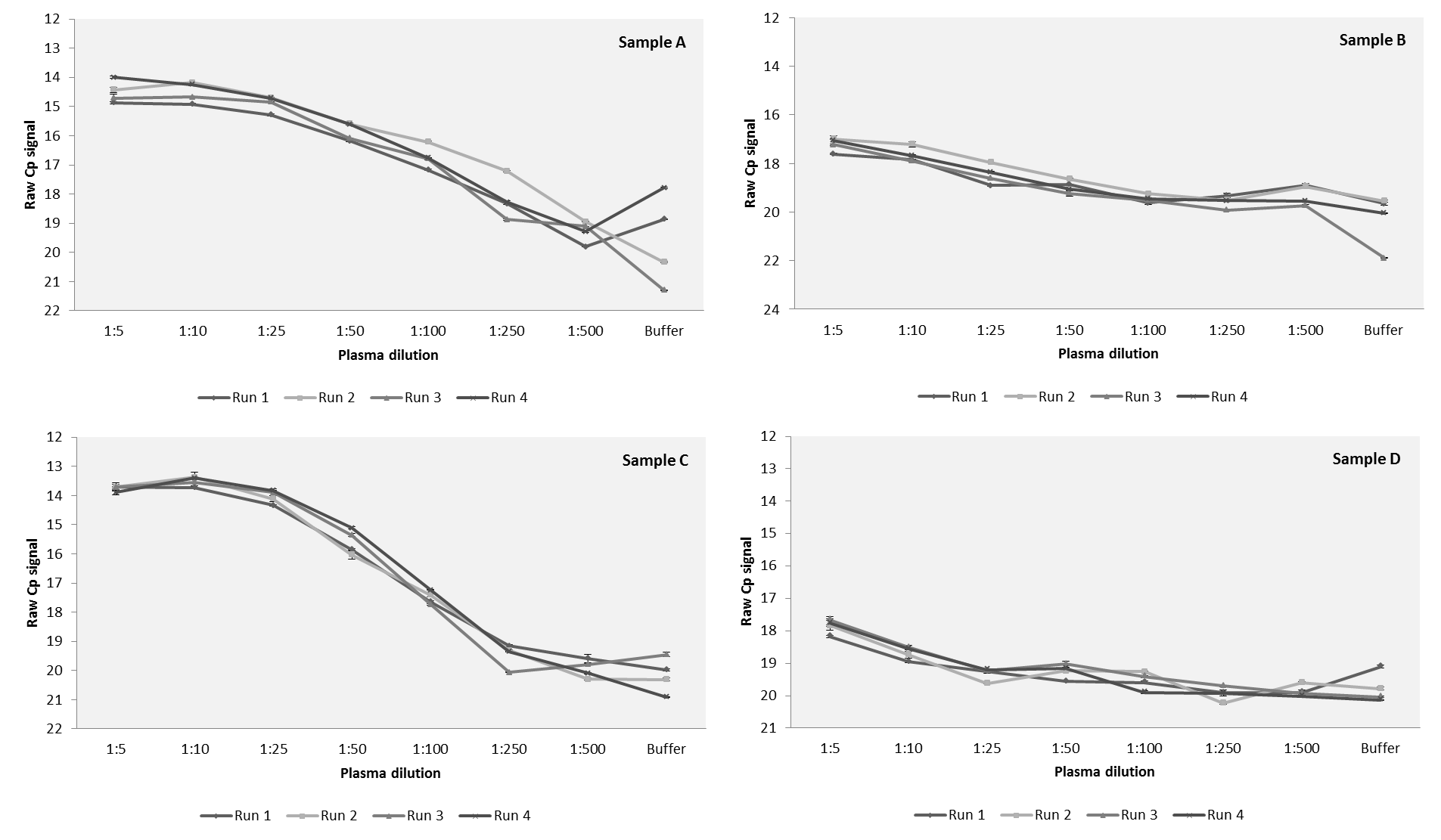

Supplement: Additional file 1 — Validation of MMP-9:TIMP-1 PLA. Figure A. Validation of MMP-9:TIMP-1 assay performance. A) Various probe concentrations. Four different proximity probe concentrations (50, 75, 100 and 200 pM) were incubated with plasma in different dilutions. The probe concentration of 50 pM demonstrated the best linear range and this was evaluated as being the optimal protocol. B) Sample and probe incubation. The difference between incubation of sample and probe mix for one hour at 37°C and ON at 4°C was tested. An increased sensitivity of 1–2 Cp values was assessed by ON incubation. C) Test for pre-amplification efficiency. To investigate if a pre-amplification was increasing the signal in the qPCR, a standard curve was run with and without pre-amplification. An increase of 15 Cp values was demonstrated due to pre-amplification illustrating high efficiency. D) Unspecific annealing of the qPCR primers. The interaction of free pre-amplification primers on the true signal was evaluated. Primer number 5 corresponds to MMP-9 and primer number 7 corresponds to TIMP-1. Only the correct 7–5 primer combination gave a true signal. The three other combinations gave only background signals. Figure B Difference between detecting the MMP-9:TIMP-1 complex with the MMP-9 (5’phosphate) and TIMP-1 (3’hydroxyl) probe combination versus the TIMP-1 (5’phosphate) and MMP-9 (3’hydroxyl) probe combination. It was found that the MMP-9 (5’phosphate) and TIMP-1 (3’hydroxyl) probe combination was performing slightly better. Figure C Dilution curves of plasma samples A-D analysed by PLA in four different days for the MMP-9:TIMP-1. These curves were used to calculate both intra- and inter-assay variation. [file 1471-2407-13-598-S1.docx]
