# Supplementary material for: Plasma levels of the MMP-9:TIMP-1 complex as prognostic biomarker in breast cancer: a retrospective study
Source: BMC Cancer. 2013 Dec 13;13:598. doi: 10.1186/1471-2407-13-598 (PMC3878682; doi:10.1186/1471-2407-13-598)
Supplement: Supplementary file 2 [file 1471-2407-13-598-S2.docx]

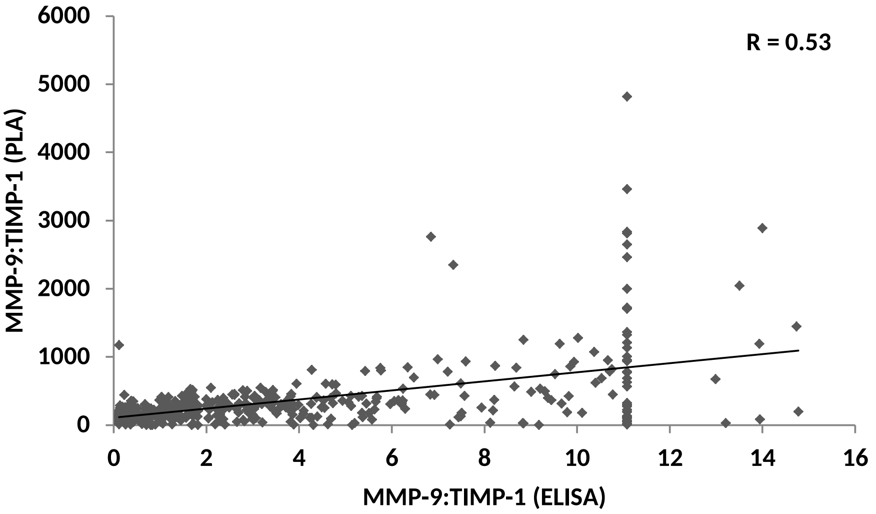
R = 0.53
MMP-9:TIMP-1 (PLA)
MMP-9:TIMP-1 (ELISA)
0
1000
2000
3000
4000
5000
6000
0
2
4
6
8
10
12
14
16
